# Migration and DNA methylation: a comparison of methylation patterns in type 2 diabetes susceptibility genes between indians and europeans

**DOI:** 10.7243/2050-0866-2-6

**Published:** 2013-02-06

**Authors:** Hannah R. Elliott, Gagandeep K. Walia, Aparna Duggirala, Alix Groom, S. Umakar Reddy, Giriraj R. Chandak, Vipin Gupta, Markku Laakso, Jacqueline M. Dekker, The RISC Consortium, Mark Walker, Shah Ebrahim, George Davey Smith, Caroline L. Relton

**Affiliations:** 1 Institute of Genetic Medicine, Newcastle University, Newcastle-upon-Tyne, UK; 2 South Asia Network for Chronic Disease, Public Health Foundation of India, New Delhi, India; 3 Centre for Cellular and Molecular Biology, Council of Scientific and Industrial Research, Hyderabad, India; 4 University of Eastern Finland, Finland, and Kuopio University Hospital, Finland; 5 Department of Epidemiology and Biostatistics, EMGO Institute for Health and Care Research, VU University Medical Centre, Amsterdam, the Netherlands; 6 See Supplementary text; 7 Institute of Cellular Medicine, Newcastle University, Newcastle-upon-Tyne, UK; 8 Department of Epidemiology and Population Health, London School of Hygiene and Tropical Medicine, London, UK; 9 MRC Centre for Causal Analyses in Translational Epidemiology, Department of Social and Community Medicine, University of Bristol, Bristol, UK

**Keywords:** Type 2 diabetes, DNA methylation, ethnicity

## Abstract

**Background:**

Type 2 diabetes is a global problem that is increasingly prevalent in low and middle income countries including India, and is partly attributed to increased urbanisation. Genotype clearly plays a role in type 2 diabetes susceptibility. However, the role of DNA methylation and its interaction with genotype and metabolic measures is poorly understood. This study aimed to establish whether methylation patterns of type 2 diabetes genes differ between distinct Indian and European populations and/or change following rural to urban migration in India.

**Methods:**

Quantitative DNA methylation analysis in Indians and Europeans using Sequenom^®^ EpiTYPER^®^ technology was undertaken in three genes: ADCY5, FTO and KCNJ11. Metabolic measures and genotype data were also analysed.

**Results:**

Consistent differences in DNA methylation patterns were observed between Indian and European populations in ADCY5, FTO and KCNJ11. Associations were demonstrated between FTO rs9939609 and BMI and between ADCY5rs17295401 and HDL levels in Europeans. However, these observations were not linked to local variation in DNA methylation levels. No differences in methylation patterns were observed in urban-dwelling migrants compared to their non-migrant rural-dwelling siblings in India.

**Conclusions:**

Analysis of DNA methylation at three type 2 diabetes susceptibility loci highlighted geographical and ethnic differences in methylation patterns. These differences may be attributed to genetic and/or region-specific environmental factors.

## Background

Type 2 diabetes represents a major global health burden that is particularly increasing in prevalence in low and middle income countries, including India. Urbanisation is one reason attributed to this increase. Comparison of rural and urban dwelling Indians has uncovered increases in body mass index (BMI) and other metabolic traits associated with type 2 diabetes in urban dwellers [1,2].

Epigenetic variation, including DNA methylation, is involved in the mechanisms of gene regulation and has become a focus of interest in the domain of common complex disease [3]. There is increasing evidence that methylation levels change with age and with environmental exposure(s) [4–6]. Additionally, underlying genetic variation also plays a role in determining patterns of DNA methylation [7–11]. Population-level differences in genetic architecture may therefore reflect differences in DNA methylation patterns between populations. Furthermore, these patterns may also relate to differential disease susceptibility [12,13].

DNA methylation was analysed in three genes, ADCY5, FTO and KCNJ11. These genes were selected as they are established type 2 diabetes susceptibility genes that are plausibly regulated by epigenetic mechanisms: each locus contains dense regions of methylation (CpG) sites, known as CpG islands, close to the gene promoter and binding sites for methylation sensitive regulators of transcription [14]. Increased DNA methylation on the FTO obesity susceptibility haplotype [15], hypomethylation of FTO in type 2 diabetes cases compared to controls [16] and evidence (although not demonstrating a causal relationship) that the effect of the FTO rs9939609 risk allele may be mediated by methylation changes in other genes [17] have all been described in human peripheral blood DNA. DNA methylation in ADCY5 is associated with decreased gene expression in leukemia patients [18], also showing evidence for regulation of this gene by methylation. Evidence for variable KCNJ11 methylation has not yet been published, although methylation of other K_ATP_ channel subunits has recently been described in mice and has been postulated to regulate gene expression [19].

Additionally, these genes were selected on the basis that they are associated with type 2 diabetes or related traits in both Indian and European populations. FTO has well replicated associations with type 2 diabetes in numerous populations including those in India and Europe [20–23]. The association between type 2 diabetes and genetic variants in KCNJ11 has also been identified in both populations [24–27]. Variants in ADCY5 associate with fasting and 2-hour plasma glucose levels following oral glucose tolerance tests in Europeans [28,29] and in Indians [30].

This study sought to identify differences in DNA methylation between Indians and Europeans and to assess the relationship between DNA methylation and underlying SNP architecture at three type 2 diabetes susceptibility loci. It also aimed to identify rural-urban migration-related differences in DNA methylation patterns at the loci analysed and to assess the relationship between DNA methylation and migration-induced shifts in metabolic traits in Indian subjects. Use of geographically distinct populations from North and South India allowed differences in methylation patterns within India to be determined.

## Methods

### Study populations

#### Indian Migration Study (IMS)

The IMS was conducted in four factories located in cities across India: Lucknow (Northern), Nagpur (Central), Hyderabad (Southern) and Bangalore (Southern). Factory workers and their co-resident spouses were recruited to the study if they were rural-urban migrants. Non-migrant siblings living in the rural village of origin were also invited to join the study. If migrants had multiple siblings, they were asked to invite the one closest to them in age and of the same sex. The fieldwork for this study took place between 2005 and 2007. In-depth study design details and preliminary findings from the IMS have been reported elsewhere [1,31–33]. This report studies a subset of IMS participants: 92 rural-urban sibling pairs recruited from Hyderabad and 92 rural-urban sibling pairs recruited from Lucknow. Criteria for selecting sibling pairs were that they were of the same sex, age matched (within 5 years) and had provided DNA samples. Siblings are referred to throughout as ‘urban’ and ‘rural’ samples depending on their migration status. The sub-group were aged between 22 and 66 years, with an average age of 45.8 (SD= 7.3). The mean time since migration was 26.5 years.

#### Relationship between insulin sensitivity and cardiovascular disease (RISC) cohort

This cohort is a collection of healthy Europeans (white Caucasian) aged between 30 and 60 years, recruited from 19 centres in 14 countries across Europe. Data were collected between 2002 and 2004. The recruitment methods of the RISC study have been described previously [34]. This report studies a subset of 351 RISC participants with a mean age of 44.6 years (SD= 8.3) who were selected randomly from those who had fasting glucose levels of ≤7 mmol/L, provided DNA samples and underwent euglycaemic-hyperinsulinaemic clamp measurements.

### Measurements

#### Metabolic and anthropometric measures

In RISC, fasting blood samples collected at each study centre were separated into plasma and serum, aliquoted and stored (-20°C for plasma glucose, -80°C for lipids and insulin). Samples were transported to central laboratories for analysis. Height was measured using a clinical stadiometer. Body weight was measured using a bioimpedance balance (Tanita International Division, UK).

In IMS, fasting blood samples were collected and separated into plasma and serum. Fasting plasma glucose was measured on the day of collection at each of the four study centres. Serum insulin and lipids were analysed at the All India Institute of Medical Sciences, New Delhi, India. Height was measured using a portable plastic stadiometer. Body weight was measured using digital weighing scales with 100g accuracy.

In both studies, BMI was calculated as a function of weight (kg)/height (m^2^). Homeostasis model assessment-insulin resistance (HOMA-IR) was calculated as: (fasting plasma glucose (mmol/L) x fasting serum insulin (mU/L))/22.5.

#### DNA methylation

In RISC, DNA was extracted from whole blood using a Nucleon^®^ BACC2 kit according to manufacturer’s instructions. In IMS, DNA was extracted from whole blood using a salt precipitation method. Methylation analysis was performed separately at each centre, both following identical protocols.

Genomic DNA (1 µg) was bisulphite modified using an EZ DNA Methylation kit (Zymo Research, CA, USA). The manufacturer’s protocol was followed with modifications (as recommended by Sequenom^®^). Cycling conditions were adjusted to 20 cycles of 95°C for 30 seconds and 50°C for 15 minutes. Column spin times were doubled, and final elution volume was 100 µL.

**Figure 1A-C.**
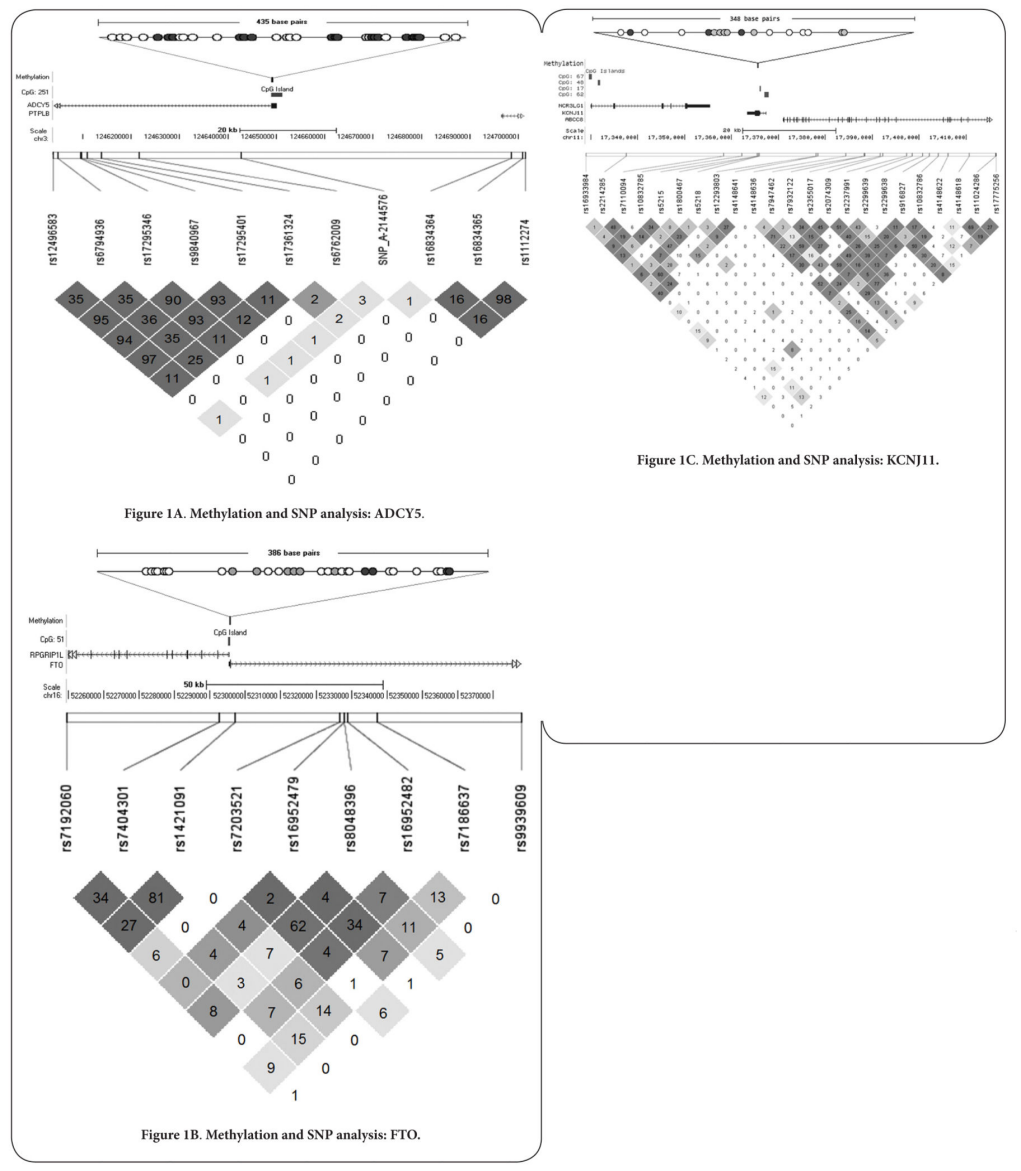
Figures show the region of the chromosome that methylation was measured in and location of SNPs analysed. Upper images show the amplicons across which methylation was measured. Each circle represents a CpG site in the amplicon analysed. Dark filled circles represent CpG sites from which the average methylation value for the amplicon was generated. Lower images show linkage disequilibrium between SNPs. Values in diamonds are r^2^ values. Diamond colours represent LOD and D’ (white: D’<1, LOD <2; dark grey: D’= 1, LOD≥ 2; shades of grey:D’<1, LOD≥ 2).

Assays were designed using EpiDesigner software (Sequenom^®^) and methylation analysis was conducted using the Sequenom^®^ EpiTYPER^®^ according to the Sequenom^®^ protocol. Amplicons were designed to capture the largest number of CpG sites possible within or close to CpG islands at each of the three loci investigated. Assays for ADCY5 and FTO loci were located within CpG islands while the amplicon designed for KCNJ11 was 221 base pairs upstream of the nearest CpG island. Further details regarding the CpG sites measured are shown in Figures 1A-C. Oligonucleotide sequences are available from the authors on request. Methylation data were generated as β values between 0 and 1, indicating percentage methylation of the original template.

Methylation data were generated at least in duplicate. Methylation for a small number of CpG sites at each locus could not be measured as the fragment masses were beyond the range for MALDI-TOF detection (ADCY5: n= 3; FTO: n= 9; KCNJ11: n= 1). CpG sites with overlapping or duplicated mass fragments following MALDI-TOF were excluded from analysis (ADCY5: n= 9; FTO: n= 7; KCNJ11: n= 4). Replicates returning methylation values that were more than 10% discordant were also excluded. Average methylation values were calculated from concordant measures. CpG sites with concordant data for ≤80% of individuals were excluded (ADCY5: n= 4; FTO: n= 3; KCNJ11: n= 2). CpG sites with median methylation levels in both IMS and RISC cohorts with values of ≤2% or ≥98% were also excluded from the analysis (FTO: n= 6; KCNJ11: n= 7).

Of the remaining CpG sites (ADCY5: n= 17; FTO: n= 4; KCNJ11: n= 3), correlation of DNA methylation was demonstrated across all CpG sites within each gene (ADCY5: r= 0.66, p= 8.500e-24; FTO: r= 0.51, p= 4.996e-44, KCNJ11: r= 0.23, p= 8.857e-06). Average methylation at each locus was calculated for each study participant and utilised for analysis. Analyses involving non-combined values were not materially different and are not provided here.

#### Genotyping

GWAS data had previously been generated from the RISC participants using an Affymetrix Genome-wide Human SNP Array 6.0 (Affymetrix, USA). Genotypes for SNPs within 50kb of the regions analysed for methylation were extracted from the GWAS data set (FTO: n= 17; ADCY5: n= 36; KCNJ11: n= 42). Additionally, Affymetrix FTO genotype data for SNP rs9939609 was included in the analysis as this SNP is associated with BMI and diabetes in GWAS. This SNP lies approximately 82kb from the region in FTO where methylation was measured. SNPs that were not in Hardy-Weinberg equilibrium (p<0.05) or had a minor allele frequency of <5% were excluded. SNPs that were adequately tagged by other SNPs were also excluded. Tagging SNPs were identified via the tagger function in Haploview using pairwise tagging and an r^2^ threshold of 0.8 [35]. Nine FTO, seven ADCY5 and 23 KCNJ11 SNPs were included in the final analysis.

In the IMS, genotyping of FTO SNP rs9939609 had previously been conducted using the Sequenom^®^ MassARRAY™.

### Ethical approval

Ethical approval for the IMS was obtained from an Indian central institutional review board (All India Institute of Medical Sciences (AIIMS), New Delhi, India (Reference Number: A-60/4/8/2004), as well as institutional review boards at each of the study sites. Written informed consent (witnessed thumbprint if illiterate) was obtained from the participants.

For the RISC study, local ethics committee approval was obtained by each recruitment centre. Written informed consent was obtained from all participants.

### Statistical Analysis

Methylation and metabolic data were not normally distributed in the data sets, therefore non-parametric tests for association between groups were used. When measuring any effect of rural to urban migration, paired analysis was utilised. When comparing groups within India (Hyderabad and Lucknow) and between Indians and Europeans, analyses were performed separately across rural and urban individuals using unpaired tests. Europeans, who were considered to be urban dwellers, were compared to urban Indian sub-groups only. Age was not different between analysis groups and stratification by sex did not alter results reported.

The RISC cohort included healthy individuals with fasting glucose levels of ≤7 mmol/L. However, the IMS cohort included a number of individuals with type 2 diabetes (n= 34) and individuals without diagnosed type 2 diabetes but fasting glucose levels of >7 mmol/L (n= 2). Removal of these 36 individuals from the IMS group had no appreciable effect on any of the analyses presented; they are included in the analyses described here.

Linear regression analysis was conducted to investigate the relationship between methylation and metabolic measures. Diagnostic tests indicated that residuals were normally distributed and homoscedastic.

Where multiple SNPs were analysed, data was adjusted for multiple testing using a Bonferroni correction of unadjusted p-values. The denominator in the Bonferroni correction was determined as the sum of the LD blocks plus the number of singleton SNPs (SNPs not in a block). To define blocks genotyping in RISC participants and the Solid Spine of LD (SSLD) method as utilised in the program HAPLOVIEW was used [35]. In this method SNPs that have contiguous pairwise D’ values of ≥0.8 are included in a block. For both FTO and ADCY5, the SNPs considered in the analyses formed two blocks with two additional singleton SNPs. Consequently, the denominator in the Bonferroni correction was n= 4 at each locus. For KCNJ11, SNPs formed six LD blocks and one singleton SNP.

Where multiple metabolic measures were tested, data were also adjusted for multiple testing using a Bonferroni correction of unadjusted p-values (n= 8).

Statistical analyses were conducted using STATA 11 (STATA corporation, Texas, USA).

## Results

An overview of DNA methylation, metabolic measures and genotype in each of the study sub-groups can be found in Table 1 and Supplementary Table 1.

### Rural to urban migration

#### Methylation

Wilcoxon signed-rank tests were conducted to identify potential changes in methylation in response to urban migration. Each place (Lucknow and Hyderabad) in this paired analysis was treated independently; urban dwellers were compared pair-wise to their non-migrant siblings. No difference in average methylation in ADCY5 (Lucknow: n= 81, z= 0.683, p= 0.495; Hyderabad: n= 50, z= -1.019, p= 0.308), FTO (Lucknow: n= 88, z= 0.597, p= 0.550; Hyderabad: n= 69, z= 0.550, p= 0.582) or KCNJ11 (Lucknow: n= 83, z=-0.825, p= 0.409; Hyderabad: n= 69, z= 0.039, p=0.969) was observed.

#### Metabolic measures

Rural to urban migration in the IMS population has previously been shown to elicit changes in a range of metabolic measurements. Analysis of metabolic measures from this sub-group of the IMS population also demonstrated these changes which included increased BMI and HOMA-IR in urban groups (Table 2).

### Differences Between Geographical Location

Analysis of differences between IMS urban dwelling migrants (Hyderabad and Lucknow) and RISC were performed. An overview of methylation, metabolic measures and group sizes is shown in Table 1. Statistical comparisons are described below.

#### Methylation

**Figure 2.**
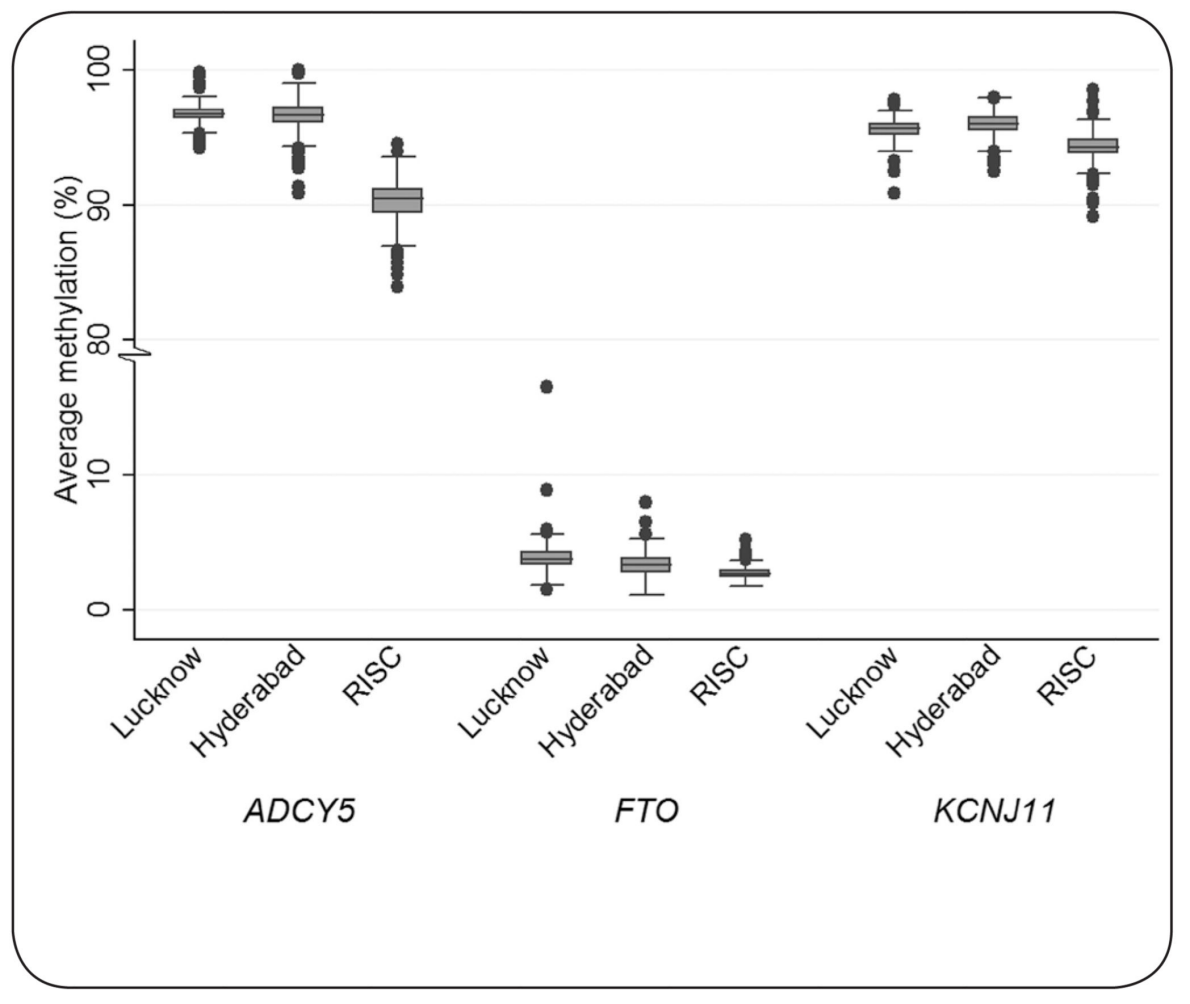
Box plot showing ADCY5, FTO and KCNJ11 methylation by place. Boxes show the median and interquartile range, whiskers represent the minimum and maximum. Closed circles are outliers.

Marked differences in methylation levels were noted between the geographic locations. ADCY5, FTO and KCNJ11 methylation was 6.3, 2.7 and 1.5% lower, respectively, in the European group compared to the Urban Indian group (Figure 2, see Table 1 for methylation values and Table 3 for test statistics). FTO methylation was 0.5% and 0.7% higher in rural and urban Lucknow dwellers compared to respective Hyderabad dwellers. Conversely, KCNJ11 methylation was 0.5% lower in Lucknow than Hyderabad, in both rural and urban dwelling comparisons. In ADCY5, methylation differences between Lucknow and Hyderabad were less marked. No robust regional differences in RISC methylation were identified between study centres (ADCY5, p= 0.362; FTO, p= 0.946; KCNJ11, p= 0.505). Furthermore, no differences in methylation were observed in RISC when Northern dwelling Europeans were compared to Southern dwelling Europeans (defined as living above or below 50° latitude) (ADCY5, p= 0.958 ; FTO, p= 0.745; KCNJ11, p= 0.637 ).

#### Metabolic measures

Several metabolic parameters differed markedly between the geographical groups (Table 3). Europeans were less insulin resistant than their Indian counterparts; HOMA-IR was on average 0.37 units lower (1.07 (0.66-1.54) vs 1.44 (0.76-2.27)). Conversely, urban Indians had a trend for lower cholesterol levels compared to Europeans. Total cholesterol was 0.17mmol/L lower in urban Indians (4.73(4.03-5.53) vs 4.90(4.30-5.40)), HDL-cholesterol was 0.21 mmol/L lower (1.19(0.98-1.40) vs 1.40(1.19-1.65)). As expected, triglycerides showed the inverse associations to HDL and were on average 0.53 mmol/L higher in urban dwelling Indians (1.46(1.13-2.05) vs 0.93(0.68-1.25)). Fasting glucose was on average 0.40 mmol/L higher in Lucknow than Hyderabad, in both urban (5.55(4.94-6.27) vs 5.05(4.72-5.27)) and rural groups (5.22(4.76-5.80) vs 4.94(4.66-5.16)). No differences were observed in total cholesterol. In general, it was noted that metabolic differences between Hyderabad and RISC were smaller than between Lucknow and RISC groups (see Table 1 and 3 for details). Stratification by age or sex did not alter the outcome of these analyses.

### SNP architecture: Europeans

SNP genotypes were available for the European RISC cohort.Additionally, rs9939609 genotype data were available for the IMS cohort. SNPs were analysed to investigate whether: (i) local underlying genetic architecture is associated with local (*cis*) DNA methylation and (ii) local underlying genetic architecture is associated with any of the metabolic measures assessed. Selected SNPs in each gene were located within 50kb of the region that methylation levels had been measured.

### Genetic association with *cis* DNA methylation

Genotypes from a total of seven SNPs in ADCY5 were analysed (Figure 1A). When comparing ADCY5 methylation with respect to genotype using an additive model, rs17361324 and rs1112274 were associated with methylation levels. The association of SNP rs1112274 with mean ADCY5 methylation withstood correction for multiple testing (trend: z= 2.52, p= 0.012). These changes represent an approximate 0.5% increase in methylation per minor allele.

In FTO, genotypes from a total of 9 SNPs in the 50 kb region were analysed in the RISC cohort (Figure 1B). SNP rs9939609 was also analysed. When comparing FTO methylation with respect to genotype in RISC, none of the SNPs showed an association with methylation.

Genotypes from a total of 23 SNPs in KCNJ11 were analysed (Figure 1C). None of the KCNJ11 SNPs were associated with methylation at this locus.

### Genetic association with metabolic measures

When testing for association between genotype and metabolic factors, SNPs that showed association using an initial Kruskal-Wallis test were further analysed for an additive effect using a non-parametric trend test.

In ADCY5, following correction for multiple testing, one robust association remained. An increase in HDL of 0.07 mmol/L was observed when the minor allele of rs17295401 was present (trend test: z= 2.66, p= 0.008). This HDL associated SNP was not associated with DNA methylation, suggesting that the observed influence of SNP on phenotype was independent of possible effects mediated by DNA methylation.

In FTO, HDL showed a 0.16 mmol/L average increase per minor allele of rs16952479 (trend: z= 2.62, p= 0.009). Fasting insulin was on average 0.45 mU/L higher (trend: z= 2.52, p= 0.012) and BMI was on average 0.53 units higher (z= 2.82, p= 0.005) per minor allele of rs9939609.

Following correction for multiple testing, one robust association remained at the KCNJ11 locus. HDL showed a 0.16 mmol/L average decrease per minor allele of rs1800467 (trend: z= -3.33, p= 0.001).

### SNP architecture: IMS

In agreement with data generated on the RISC population, no association was seen between rs9939609 and methylation in the sub-group of the IMS used in this study. Robust association of rs9939609 with insulin or BMI was also not observed in the sub-group of the IMS. We also analysed the allele distribution of rs9939609 between the groups included in this study. When comparing urban Lucknow with RISC, there was little difference in allele frequency (Fisher’s exact p= 0.859). However, there was a robust difference in allele frequency between urban Hyderabad and RISC groups (Fisher’s exact p= 0.002). When comparing Lucknow with Hyderabad there was no difference in allele frequency between rural groups (Fisher’s exact p= 0.438) but there was a difference between urban groups (Fisher’s exact p= 0.008). This difference was being driven by an excess of minor allele homozygotes in the urban Lucknow group.

### Association between methylation and metabolic measures in Indians and Europeans

To test the hypothesis that methylation and metabolic measures might be related, regression models were constructed. Data were analysed separately for urban Hyderabad, urban Lucknow and RISC groups. Univariate regression analysis indicated that methylation in ADCY5, FTO or KCNJ11 was not robustly associated with any metabolic measure that was assessed (BMI, fasting glucose, fasting insulin, HOMA-IR, LDL, HDL, total cholesterol and triglycerides).

## Discussion

### Rural to urban migration in India

Metabolic differences between urban migrant and rural non-migrant siblings were observed, in concordance with previous observations in the whole IMS sample [1,2]. No differences in average DNA methylation were observed in any locus tested that could be ascribed to urban living. A number of factors might explain this lack of association. The loci studied may not be appropriate or sensitive biomarkers of methylation changes. Alternatively, subtle shifts in DNA methylation which might collectively exert large physiological shifts in metabolic measures may have been too small to detect with the available sample size. Post-hoc power calculations indicated an ability to detect changes in methylation of 1% (with 75% power) between rural and urban dwelling sibs at p= 0.05 and n=46; the power to detect smaller differences was limited.

The metabolic transition following migration is rapid [2] and if this is fuelled by epigenetic changes then they too must be responsive to the exogenous influences within a short period. The mean time since urban siblings had migrated was 26.5 years. It is therefore unlikely that changes in DNA methylation following migration were not observed due to the timings of sample collection. It is reasonable to postulate that methylation for these genes could have been set in early life, before migration occurred.

### Regional differences in DNA methylation within India

Methylation of FTO was consistently higher in Lucknow compared to Hyderabad samples; methylation of KCNJ11 was lower. The mean percentage difference was small (around 0.6%). These regional differences could be caused by differences in dietary habits or other lifestyle influences specific to each region. Genetic differences between groups could also be causing the differences observed. Geographical differences in methylation could therefore be explained partly by genetic population stratification and partly by population specific environmental differences.

### Geographical differences in methylation between Indian and European groups

At all loci, average methylation was markedly lower in Europeans compared to Indians. The difference in ADCY5 methylation was the largest (6.3%). Although it is recognised these differences may be caused by systematic differences in analysis between the two cohorts, any potential variability was minimised by following identical analysis protocols conducted largely by the same researchers. Furthermore, systematic differences of similar magnitude would be expected across all of the loci studied and this was not observed.

### SNP effects on metabolic traits were not mediated through change in methylation

A number of recent studies have shown that methylation patterns are to some extent linked to underlying genetic architecture [7,36,37]. We therefore tested the association between methylation levels and genotype in the European RISC cohort. Tentative evidence supporting a relationship between methylation and SNP genotype in ADCY5 was observed. Further work would allow this relationship to be confirmed both in a larger European cohort and in the IMS.

Linguistic, mitochondrial and Y-chromosome studies of North and South Indians show similarities between North Indian populations and western Eurasians [38–41]. We observed differences in rs9939609 allele frequency between RISC and Hyderabad, but not between RISC and Lucknow which supported these findings. For this reason, we had initially postulated that the Lucknow population would be more similar to RISC than Hyderabad in its metabolic and methylation profile. However, we observed that differences between Lucknow and RISC were more marked than differences between Hyderabad and RISC. This suggests that metabolic and methylation profiles might be influenced predominantly by regional environmental factors rather than by genetic factors.

### The relationship between methylation and metabolic measures.

We postulated that differences in DNA methylation in the loci tested might explain a proportion of the variation in metabolic risk. Although we identified that DNA methylation did vary between Indian and European groups, as did metabolic risk profiles, we were not able to confirm a direct relationship between DNA methylation at these loci and the metabolic measures tested. Thus the hy.3pothesis that environmentally driven changes in epigenetic patterns following migration underlie shifts in cardiometabolic disease risk remains unresolved.

### Comparison with other studies

The difference in DNA methylation between Indians and Europeans at these loci is of similar magnitude to differences described in other studies. Heijmans and colleagues [6] described altered methylation of 5.2% between periconceptional famine exposure groups in IGF2. Methylation differences in the same gene between neonates of small or appropriate birth weight for gestational age were 0.2% [42]. A study investigating methylation of pancreatic islets in type 2 diabetes patients and controls identified methylation differences of 4.4 - 9.3% between groups [43]. Methylation changes of this magnitude are known to elicit alterations in gene expression [43,44].

Other studies have also looked at the role of ethnicity in determining DNA methylation patterns. Global methylation differences of 2% have been observed between different racial groups living in North Texas, USA [45]. Another American study also described ethnic differences in DNA methylation, in this instance measuring genomic hypomethylation [46]. At the level of individual CpG sites, differences between ethnic groups have also been observed at birth [47]. Our data therefore support the limited available evidence that methylation patterns do differ between ethnic groups.

Emerging data point to the complex overlap between genotype, DNA methylation and gene expression [7,36,48]. Recent data from a Moroccan cohort identified that up to 25% of differential gene expression was explained by dwelling location even when genetic differences were small [49]. However, methylation analysis in a linked study suggested limited involvement of DNA methylation in driving these differences [50]. More work is therefore needed to further delineate these relationships.

### Further work

This pilot study identified methylation differences between two ethnically distinct cohorts at three loci. One strategy to further investigate DNA methylation differences between these cohorts would be to employ a more comprehensive genome-wide approach. To establish the relative contributions of environment and genotype, analysis in a further group of Indians who have migrated to Europe could also be of value. Assuming methylation differences exist, these approaches may identify causally related shifts in metabolic profiles and associated disease risk.

## Conclusions

This research described the analysis of DNA methylation at three distinct type 2 diabetes susceptibility loci. It highlights geographical and ethnic differences in methylation patterns, particularly between Indians and Europeans. These differences may be attributed to genetic and/or region-specific environmental factors and may help to explain the excess incidence of type 2 diabetes observed in South Asian populations.

## Supplementary Material

Supplementary Table 1

Supplementary Text

## Figures and Tables

**Table 1.** Methylation and metabolic measures.

Measure	Rural Hyderabad		Urban Hyderabad		Rural Lucknow		Urban Lucknow		Urban Indian		RISC RISC	

	n	Median (IQR)	n	Median (IQR)	n	Median (IQR)	n	Median (IQR)	n	Median (IQR)	n	Median (IQR)
**(%) *ADCY5***	63	96.6	66	96.8	83	96.7	89	96.7	155	96.8	332	90.5
**Methylation**	-	(96.0-97.0)	-	(96.3-97.0)	-	(96.4-97.3)	**-**	(96.2-97.3)	-	(96.0-97.0)	**-**	(89.4-91.2)
**(%) *FTO***	80	8.0	80	8.0	89	8.5	90	8.7	170	8.5	336	5.8
**Methylation**	-	(6.9-9.2)	**-**	(6.7-9.1)	**-**	(7.8-10.0)	**-**	(7.8-9.7)	-	(7.5-8.5)	-	(5.3-6.3)
**(%) *KCNJ11***	78	96.0	79	96.2	88	95.5	86	95.7	165	95.8	337	94.3
**Methylation**	-	(95.5-96.7)	-	(95.5-96.5)	-	(95.1-95.8)	**-**	(95.2-96.0)	-	(95.3-96.3)	-	(93.8-94.8)
**Age**	91	46	91	47	91	45	91	46	182	46	351	44
**(years)**	-	(41-52)	-	(42-51)	-	(41-50)	-	(43-50)	-	(42-50.5)	**-**	(38-51)
**Sex (% male)**	91	52%	91	52%	91	77%	91	77%	182	64%	351	43%
**Fasting glucose**	91	4.94	91	5.05	91	5.22	91	5.55	182	5.18	350	5.15
**(mmol/L)**	-	(4.66-5.16)	-	(4.72-5.27)	-	(4.76-5.80)	-	(4.94-6.27)	-	(4.83-5.72)	-	(4.80-5.50)
**Fasting insulin**	91	3.80	91	6.00	91	5.00	90	6.75	181	6.20	348	4.61
**(mU/L)**	-	(1.90-8.20)	-	(3.0-10.0)	-	(2.0-6.8)	-	(4.2-10.5)	-	(3.7-10.0)	-	(3.02-6.33)
**HOMA-IR**	80	0.84	76	1.34	84	1.03	74	1.52	150	1.44	347	1.07
	-	(0.39-1.70)	-	(0.56-2.13)	-	(0.47-1.59)	-	(0.96-2.42)	-	(0.76-2.27)	-	(0.66-1.54)
**BMI**	91	23.40	91	25.98	91	20.90	91	25.00	182	25.47	351	24.50
**(Kg/m^2^)**	-	(20.8-25.8)	-	(23.3-27.4)	-	(18.2-24.0)	-	(22.6-27.6)	-	(22.9-27.5)	-	(23.0-27.8)
**Total cholesterol**	91	4.68	91	4.68	91	4.60	90	4.78	181	4.73	348	4.90
**(mmol/L)**	-	(3.93-5.45)	-	(4.03-5.50)	-	(3.82-5.37)	-	(4.03-5.58)	-	(4.03-5.53)	-	(4.30-5.40)
**LDL**	90	2.86	91	2.93	90	2.63	90	2.73	181	2.83	345	2.90
**(mmol/L)**	-	(2.25-3.57)	-	(2.19-3.62)	-	(2.06-3.32)	-	(2.18-3.70)	-	(2.19-3.62)	-	(2.40-3.50)
**HDL**	91	1.09	91	1.14	91	1.21	90	1.21	181	1.19	348	1.40
**(mmol/L)**	-	(0.96-1.29)	-	(0.56-1.37)	-	(1.03-1.47)	-	(1.03-1.40)	-	(0.98-1.40)	-	(1.19-1.65)
**Triglycerides**	91	1.36	91	1.41	91	1.32	90	1.57	181	1.46	348	0.93
**(mmol/L)**	-	(1.07-1.92)	-	(1.06-2.04)	-	(0.92-1.90)	-	(1.22-2.14)	-	(1.13-2.05)	-	(0.68-1.25)

**Table 2.** Paired analysis of metabolic measure differences between urban and rural siblings. Table shows the number of pairs of samples in each test (**n**), the test statistic (**z**) and unadjusted (**p**) value for each test. Difference (**diff**) is the median (**IQR**) of the differences between pairs of samples for each metabolic measure where rural dwellers are the reference group.

Measure	Wilcoxon signed-rank test statistics. Rural to urban comparisons	
	Lucknow	Hyderabad
Fasting glucose (mmol/L)	n=91	n=91
	z= 3.838	z= 1.598
	*p*= 0.124e-03	*p*= 0.110
	diff= 0.30 (-0.11-1.00)	diff= 0.11 (-0.22-0.50)
Fasting insulin (mU/L)	n= 90	n= 91
	z= 4.535	z= 2.767
	*p*= 5.765e-06	*p*= 0.006
	diff= 2.35(-0.40-5.10)	diff= 1.00(-1.30-4.30)
HOMA-IR	n= 71	n= 71
	z= 4.332	z= 2.659
	*p*= 0.0148e-03	*p*= 0.008
	diff= 0.60(-0.18-1.15)	diff= 0.28(-0.25-1.03)
BMI (Kg/m^2^)	n= 91	n= 91
	z= 6.345	z= 4.908
	*p*= 2.231e-10	*p*= 9.210e-07
	diff= 4.04(0.64-7.18)	diff= 1.94(-0.20-5.44)
Total cholesterol (mmol/L)	n= 90	n= 91
	z= 1.722	z= 0.267
	*p*= 0.085	*p*= 0.789
	diff= 0.23(-0.78-1.27)	diff= -0.05(-0.67-0.72)
LDL (mmol/L)	n= 89	n= 90
	z= 1.471	z= -0.026
	*p*= 0.141	*p*= 0.979
	diff= 0.22(-0.67-1.22)	diff= 0.05 (-0.75-0.60)
HDL (mmol/L)	n= 90	n= 91
	z= -0.656	z= 1.558
	*p*= 0.512	*p*= 0.119
	diff= -0.01(-0.18-0.18)	diff= 0.05(-0.10-0.21)
Triglycerides (mmol/L)	n= 90	n= 91
	z= 2.438	z= 1.181
	*p*= 0.015	*p*= 0.237
	diff= 0.31(-0.32-0.86)	diff= 0.09(-0.27-0.41)

**Table 3.** Analysis of methylation and metabolic measure by geographical location.Table shows the test statistic (*z*) and unadjusted p value for each test. Median, IQR and number of observations for each group can be found in Table 1.

Measure			Mann-Whitney U test statistics		

	Rural Hyderabad vs Rural ucknow	Urban Hyderabad vs Urban ucknow	Urban Lucknow vs RISC	Urban Hyderabad vs RISC	Urban Indian vs RISC
ADCY5	z= 2.019	z= -0.679	z= 14.493	z= 12.728	z= 17.722
methylation	p= 0.044	p= 0.497	p= 1.341e-47	p= 4.152e-37	p= 2.831e-70
FTO	z= 2.719	z= 3.198	z= 13.169	z= 10.688	z= 15.435
methylation	p= 0.007	p= 0.001	p= 1.323e-39	p= 1.159e-26	p= 9.495e-54
KCNJ11	z= -4.266	z= -3.419	z= 11.474	z= 11.109	z= 14.601
methylation	p= 1.991e-05	p= 6.279e-04	p= 1.780e-30	p= 1.131e-28	p= 2.787e-48
Fasting glucose	z= 3.292	z= 4.921	z= 4.873	z= -2.108	z= 1.779
(mmol/L)	p= 0.001	p= 8.608e-07	p= 1.100e-06	p= 0.035	p= 0.075
Fasting insulin	z= 0.365	z= 1.239	z= 4.836	z= 2.892	z= 4.972
(mU/L)	p= 0.715	p= 0.216	p= 1.324e-06	p= 0.004	p= 6.633e-07
HOMA-IR	z= 0.484	z= 1.517	z= 4.220	z= 1.797	z= 3.909
	p= 0.629	p= 0.129	p= 0.024e-03	p= 0.072	p= 0.093e-03
BMI	z= -3.550	z= -1.102	z= -0.128	z= 1.515	z= 0.893
(Kg/m^2^)	p= 0.385e-03	p= 0.271	p= 0.898	p= 0.130	p= 0.372
Total	z= -0.519	z= 0.153	z= -0.576	z= -0.921	z= -0.964
cholesterol (mmol/L)	p=0.604	p=0.878	p=0.565	p=0.357	p=0.335
LDL	z= -1.628	z= -0.621	z= -0.943	z= -0.238	z= -0.758
(mmol/L)	p= 0.104	p= 0.534	p= 0.346	p= 0.812	p= 0.448
HDL	z= 3.107	z= 1.782	z= -4.871	z= -6.654	z= -7.425
(mmol/L)	p= 0.002	p= 0.075	p= 1.109e-06	p= 2.849e-11	p= 1.127e-13
Triglycerides	z= -0.899	z= 1.311	z= 8.147	z= 7.347	z= 9.975
(mmol/L)	p= 0.369	p= 0.190	p= 3.741e-16	p= 2.022e-13	p= 1.963e-23
