# Supplementary material for: Migration and DNA methylation: a comparison of methylation patterns in type 2 diabetes susceptibility genes between indians and europeans
Source: J Diabetes Res Clin Metab. Author manuscript; Available in PMC 2016 Apr 18. (PMC4835020; doi:10.7243/2050-0866-2-6)
Supplement: Supplementary Table 1 [file NIHMS67583-supplement-1.pdf]

Supplementary Table 1| *ADCY5*, *FTO* and *KCNJ11* SNPs

**Locus**	**SNP**	**Urban Hyderabad (n)**	**Minor Allele Frequency (%)**	**Urban Lucknow (n)**	**Minor Allele Frequency (%)**	**RISC (n)**	**Minor Allele Frequency (%)**
ADCY5	rs6794936					320	48
	rs17295401					320	29
	rs17361324					318	22
	rs6762009					320	14
	rs41435846					320	36
	rs16834364					319	31
	rs1112274					320	7
FTO	rs7192060					320	13
	rs7404301					320	29
	rs1421091					320	33
	rs7203521					315	39
	rs16952479					320	4
	rs8048396					320	45
	rs16952482					265	9
	rs7186637					318	18
	rs9939609	89	28	89	42	248	41
KCNJ11	rs16933984					320	19
	rs2214285					319	7
	rs7110094					310	12
	rs10832785					320	38
	rs5215					318	37
	rs1800467					289	6
	rs5218					312	29
	rs12293803					320	7
	rs4148641					313	24
	rs4148636					304	11
	rs7947462					317	27
	rs7932122					314	10
	rs2355017					320	26
	rs2074309					320	41
	rs2237991					317	26
	rs2299639					308	20
	rs2299638					320	16
	rs916827					320	44
	rs10832786					320	14
	rs4148622					312	29
	rs4148618					320	20
	rs11024286					317	32
	rs17775256					320	34
