# Supplementary material for: Migration and DNA methylation: a comparison of methylation patterns in type 2 diabetes susceptibility genes between indians and europeans
Source: J Diabetes Res Clin Metab. Author manuscript; Available in PMC 2016 Apr 18. (PMC4835020; doi:10.7243/2050-0866-2-6)
Supplement: Supplementary Text [file NIHMS67583-supplement-2.pdf]

Appendix: RISC INVESTIGATORS

RISC recruiting centres
Amsterdam, The Netherlands: RJ Heine, J Dekker, S de Rooij, G Nijpels, W Boorsma
Athens, Greece: A Mitrakou, S Tournis, K Kyriakopoulou, P Thomakos
Belgrade, Serbia: N Lalic, K Lalic, A Jotic, L Lukic, M Civcic
Copenhagen, Denmark: J Nolan
Dublin, Ireland: TP Yeow, M Murphy, C DeLong, G Neary, MP Colgan, M Hatunic
Frankfurt, Germany: T Konrad, H Böhles, S Fuellert, F Baer, H Zuchhold
Geneva, Switzerland: A Golay, E Harsch Bobbioni,V. Barthassat, V. Makoundou, TNO Lehmann, T Merminod
Glasgow, Scotland: JR Petrie (, C Perry, F Neary, C MacDougall, K Shields, L Malcolm
Kuopio, Finland: M Laakso, U Salmenniemi, A Aura, R Raisanen, U Ruotsalainen, T Sistonen, M Laitinen, H Saloranta
London, England: SW Coppack, N McIntosh, J Ross, L Pettersson, P Khadobaksh
Lyon, France: M Laville, F. Bonnet (now Rennes), A Brac de la Perriere, C Louche-Pelissier, C Maitrepierre, J Peyrat, S Beltran, A Serusclat
Madrid, Spain: R. Gabriel, EM Sánchez, R. Carraro, A Friera, B. Novella
Malmö, Sweden (1): P Nilsson, M Persson, G Östling, (2): O Melander, P Burri
Milan, Italy: PM Piatti, LD Monti, E Setola, E Galluccio, F Minicucci, A Colleluori
Newcastle-upon-Tyne, UK: M Walker, IM Ibrahim, M Jayapaul, D Carman, C Ryan, K Short, Y McGrady, D Richardson
Odense, Denmark: H Beck-Nielsen, P Staehr, K Hojlund, V Vestergaard, C Olsen, L Hansen
Perugia, Italy: GB Bolli, F Porcellati, C Fanelli, P Lucidi, F Calcinaro, A Saturni
Pisa, Italy: E Ferrannini, A Natali, E Muscelli, S Pinnola, M Kozakova, A Casolaro, BD Astiarraga
Rome, Italy: G Mingrone, C Guidone, A Favuzzi. P Di Rocco
Vienna, Austria: C Anderwald, M Bischof, M Promintzer, M Krebs, M Mandl, A Hofer, A Luger, W Waldhäusl, M Roden

Project Management Board: B Balkau (Villejuif, France), SW Coppack (London, England), JM Dekker (Amsterdam, The Netherlands), E Ferrannini (Pisa, Italy), A Golay (Geneva, Switzerland), A Mari (Padova, Italy), A Natali (Pisa, Italy), J Petrie (Dundee, Scotland), M Walker (Newcastle, England)

Core laboratories and reading centres
Lipids Dublin, Ireland: P Gaffney, J Nolan, G Boran
Hormones Odense, Denmark: C Olsen, L Hansen, H Beck-Nielsen
Albumin:creatinine Amsterdam, The Netherlands: A Kok, J Dekker
Genetics Newcastle-upon-Tyne, England: S Patel, M Walker
Stable isotope laboratory Pisa, Italy: A Gastaldelli, D Ciociaro
Adiponectin, CRP , MBL Odense, Denmark: Allan Flyvbjerg
Ultrasound reading centre Pisa, Italy: M Kozakova
ECG reading, Villejuif, France: MT Guillanneuf
Actigraph, Villejuif, France: B Balkau, L Mhamdi
Data Management Villejuif, France, Padova, and Pisa, Italy**:** B Balkau, A Mari, L Mhamdi, L Landucci, S Hills, L Mota
Mathematical modelling and website management Padova, Italy: A Mari, G Pacini, C Cavaggion, A Tura
Coordinating office: Pisa, Italy: SA Hills, L Landucci, L Mota
